# Early-Onset 15q11.2 Microdeletion Syndrome in a Six-Year-Old Child: A Case Report of Refractory Epilepsy, Autism, and Multisystem Manifestations

**DOI:** 10.7759/cureus.90530

**Published:** 2025-08-19

**Authors:** Isi Y Ortiz Hernández, Joami Noboa Rodríguez, Lisa A Bueno Fernandez, Katherine Rijo Florimon

**Affiliations:** 1 Medicine, Pontificia Universidad Católica Madre y Maestra, Santiago de los Caballeros, DOM; 2 Pediatric Neurology, Hospital Regional Infantil Dr. Arturo Grullón, Santiago de los Caballeros, DOM

**Keywords:** 15q11.2 microdeletion syndrome, autism spectrum disorder (asd), burnside-butler syndrome, case report, medication-refractory epilepsy, multisystemic comorbidities, neurodevelopmental disorders, pediatric neurodevelopment, rare genetic disorder, schizencephaly

## Abstract

The 15q11.2 microdeletion syndrome, also known as Burnside-Butler syndrome (BBS), is a rare genetic disorder involving a deletion in the breakpoint 1 to breakpoint 2 (BP1-BP2) on the long arm of chromosome 15, often associated with growth retardation and delayed speech development. In contrast, rare manifestations consist of dysmorphic traits, seizures, and neurodevelopmental or psychiatric conditions such as epilepsy, autism spectrum disorder (ASD), and schizophrenia. The BP1-BP2 region contains genes critical for brain development and function, including non-imprinted in Prader-Willi/Angelman syndrome 1 (NIPA1), non-imprinted in Prader-Willi/Angelman syndrome 2 (NIPA2), cytoplasmic FMR1 interacting protein 1 (CYFIP1), and tubulin gamma complex associated protein 5 (TUBGCP5), which have been linked to conditions such as attention-deficit/hyperactivity disorder (ADHD), obsessive-compulsive disorder (OCD), and epilepsy. Prenatal tests and karyotype lead to unclear results, but the current chromosomal microarray analysis (CMA) provides an accurate diagnosis of BBS. Treatment for these individuals is personalized and typically involves a multidisciplinary approach. We present the case of a six-year-old male patient with 15q11.2 microdeletion syndrome and a complex neurological and developmental profile, including developmental delay and ASD. We highlight the rare combination of early-onset refractory epilepsy, schizencephaly, and cystic fibrosis transmembrane conductance regulator (CFTR) variant carrier status, which adds to the uniqueness of the case. This article contributes to expanding the clinical spectrum of 15q11.2 microdeletion syndrome. It underscores the importance of genetic testing in children with complex neurodevelopmental symptoms, as the clinical presentation of this syndrome is often subtle or nonspecific, making early diagnosis and genetic counseling challenging but essential for guiding appropriate interventions.

## Introduction

The 15q11.2 microdeletion syndrome, also known as Burnside-Butler syndrome (BBS), is a rare genetic disorder caused by the loss of genetic material on the long arm of chromosome 15, specifically in the 11.2 region. The most common features include growth retardation and delayed speech development. In contrast, rare manifestations include dysmorphic traits, seizures, and neurodevelopmental or psychiatric conditions such as epilepsy, autism spectrum disorder (ASD), and schizophrenia. The long arm of chromosome 15 contains five breakpoints, designated breakpoint 1 to breakpoint 5 (BP1-BP5). Breakpoints are specific regions of the chromosome where the DNA sequence is highly similar, making them prone to breaks, which can lead to genetic instability. Deletions in the BP1-BP2 region occur in approximately 0.5%-1% of the population. The syndrome is autosomal dominant with incomplete penetrance. Penetrance refers to the likelihood that a person carrying a specific genetic variant will express the associated trait or condition. In this syndrome, penetrance is incomplete, meaning that many carriers may show few or no symptoms. This results in a highly variable presentation, often mild, as roughly 90% of carriers may show little to no clinical symptoms, making diagnosis and genetic counseling particularly challenging. While prenatal screening and karyotype lead to unclear results, current diagnostic techniques for this syndrome utilize chromosomal microarray analysis (CMA) [1-3]. This case report illustrates the phenotypic variability associated with 15q11.2 microdeletion syndrome (BP1-BP2) in a six-year-old male. Specifically, we highlight the rare combination of early-onset refractory epilepsy, schizencephaly, a rare congenital brain malformation in which a cerebrospinal fluid-filled cleft, lined with gray matter, extends from the brain surface to the ventricle, and cystic fibrosis transmembrane conductance regulator (CFTR) variant carrier status, which adds to the uniqueness of the case [4]. By documenting this constellation of findings, the report contributes to expanding the clinical spectrum of the 15q11.2 microdeletion syndrome.

## Case presentation

We present the case of a six-year-old male patient with a complex, heterogeneous, and multisystemic clinical presentation, who was diagnosed with 15q11.2 microdeletion syndrome. The patient was delivered via cesarean section at 38 weeks of gestation, with a birth weight of 6.15 pounds and no perinatal complications. The pregnancy was unplanned but uneventful, with no maternal illnesses or teratogenic exposures; the mother took folic acid supplements before and during pregnancy and had previously used oral contraceptives. There is no reported parental consanguinity.

Horizontal and vertical nystagmus was observed from birth and initially reported by the mother. At eight days of life, during a routine pediatric visit, the nystagmus was confirmed. Ophthalmologic evaluation revealed nystagmus and strabismus. Initial neuroimaging with brain computed tomography (CT) revealed an intracranial lesion. Followed by contrast-enhanced magnetic resonance imaging (MRI) at one month of age, the lesion was found to be a cystic formation communicating with the temporal horn of the right lateral ventricle. Neurosurgical evaluation deemed the lesion operable, the first tumor resection was performed at nine months of age, and a red blood cell transfusion was performed before surgery due to anemia, with a hemoglobin level of 8 g/dL and hematocrit of 28%. Postoperatively, the mother noted improved social engagement, including smiling; however, by 12 months, the patient experienced a recurrence of nystagmus. The child began having generalized tonic-clonic seizures at one year of age, but only started treatment at 2.5 years. In 2023, the seizures proved resistant to multiple medications, raising suspicion of a genetic condition by the pediatric neurologist, and the patient was referred to a pediatric geneticist. Genetic whole-exome sequencing was performed in 2024, leading to the diagnosis of 15q11.2 microdeletion syndrome.

The patient's seizure history began at nine days of life during a respiratory infectious process, which required admission to the neonatology unit. Since then, electroencephalograms have shown normal results, but the patient has experienced refractory epilepsy with diverse semiological presentations. These include generalized tonic-clonic seizures, absence seizures, somatosensory seizures, and autonomic seizures (episodes of profuse sweating), which the International League Against Epilepsy (ILAE) recognizes as focal non-motor onset seizures [5]. The mother reported that following vagus nerve stimulator (VNS) implantation at five years of age, the seizure frequency gradually decreased over five months, declining from 10 to 12 daily episodes to two to three episodes per day, representing an approximate 77% reduction. His epilepsy is resistant to levetiracetam, valproic acid, and lacosamide.

**Figure 1:**
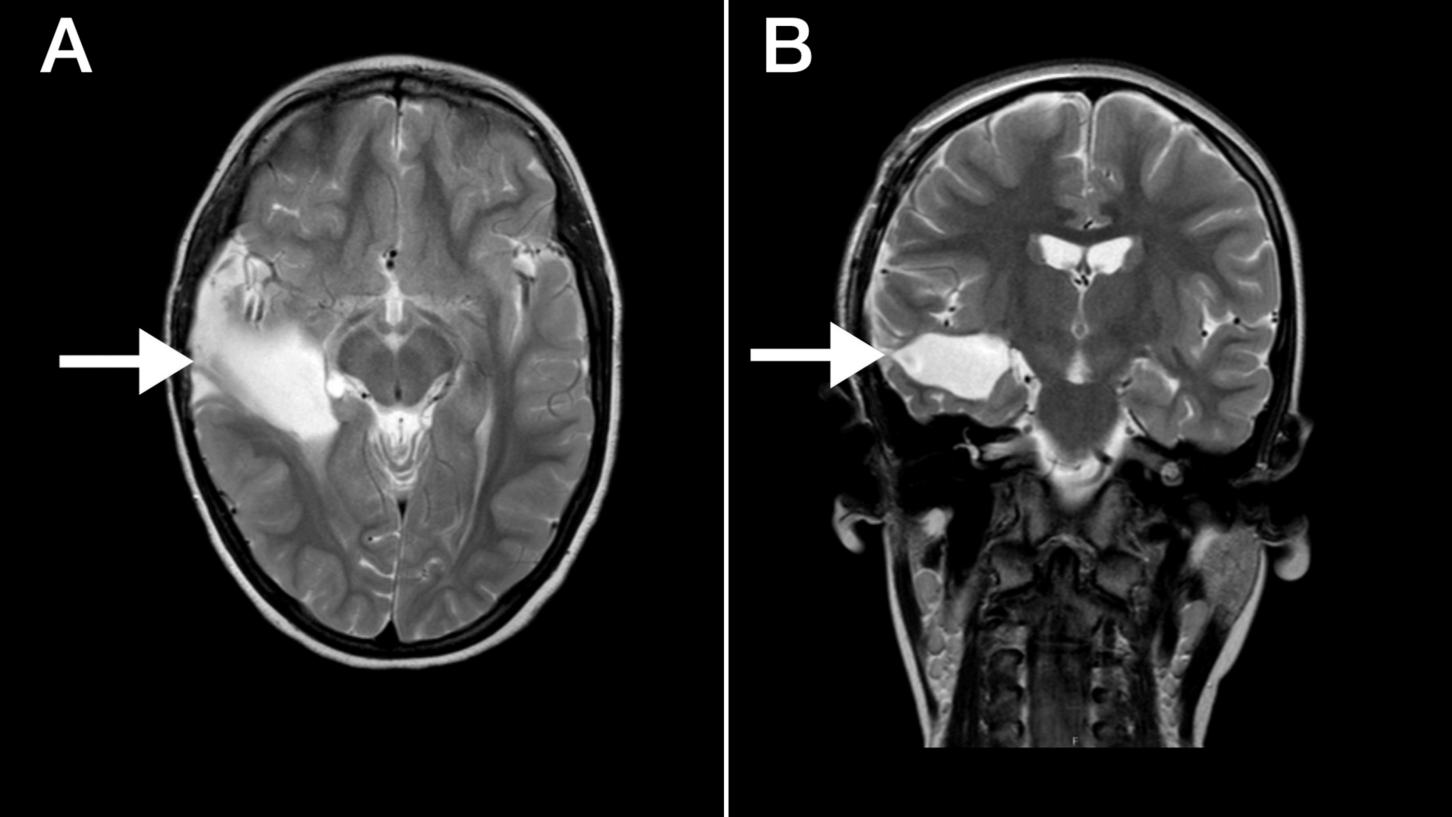
(A) Axial T2-weighted image demonstrates a hyperintense, fluid-filled cleft in the right temporal lobe communicating with the lateral ventricles (white arrow); (B) Coronal T2-weighted image confirms the fluid-filled cleft in the right temporal region communicating with the lateral ventricles (white arrow).

The patient presented with developmental regression alongside worsening frequency and intensity of generalized tonic-clonic seizures at age four. These clinical changes prompted imaging, which confirmed lesion recurrence and led to a second neurosurgical intervention. Postoperatively, the patient developed left-sided facial paralysis but has since shown gradual clinical improvement. Following a return of seizures and regression, repeat imaging confirmed lesion recurrence and led to a third neurosurgical intervention in February 2025. Histopathological analysis and glial fibrillary acidic protein (GFAP) immunoreactivity from the brain biopsy subsequently identified the lesion as a low-grade astrocytic glioma (WHO grades 1-2). The postoperative brain CT scan is shown in Figure 1, where there is a communication between the right temporal fluid-filled cleft and the ventricular system consistent with schizencephaly. A multidisciplinary team of physicians reached a consensus to consider chemotherapy; however, the decision was not unanimous. Consequently, the child was placed under observation for three months, after which a follow-up brain CT scan is planned to guide further management. Although MRI was the preferred imaging modality for follow-up, a brain CT scan was chosen due to insurance coverage limitations. During this last procedure, a VNS was implanted to control intractable seizures.

Among the neurological comorbidities identified in this patient are Level 1 ASD, diagnosed at 3.5 years, attention-deficit/hyperactivity disorder (ADHD) at five years, and schizencephaly, identified on brain MRI during the neonatal period. Behavioral manifestations include insomnia, poor eye contact, self-injurious behavior, aggression beginning at age two, speech delay with regression noted at age five, food selectivity with a highly restrictive diet, and auditory hypersensitivity with normal tympanometry. At the time of evaluation, psychiatric management included hydroxyzine and risperidone, though the mother reported persistent symptoms and worsening aggression. Motor development milestones were delayed: head control at four months, sitting without support at 10 months, and walking at 18 months. Despite significant challenges, the patient is toilet-trained and independent with toileting.

Additional comorbidities include asthma, allergic rhinitis, atopic dermatitis, drug allergies (such as penicillin, nonsteroidal anti-inflammatory drugs (NSAIDs), and propinoxate), myopia, and astigmatism. He has been diagnosed with type 2 diabetes mellitus, characterized by recurrent hyperglycemic episodes, and is currently managed without pharmacologic therapy. The patient also experiences recurrent pulmonary infections and gastrointestinal symptoms, including alternating episodes of foul-smelling diarrhea and constipation. Physical examination revealed postoperative scars: a hyperkeratotic scar on the left lateral thorax, corresponding to VNS implantation, and a hyperkeratotic scar on the right temporoparietal scalp, consistent with prior brain surgery. The pulmonary exam was unremarkable, and the cardiac examination revealed a mild systolic murmur (grade II/VI) without any pathological findings on the echocardiogram or Holter monitor. Family history is notable for epilepsy in paternal and maternal uncles; ASD diagnosed in two maternal cousins; and allergic conditions, namely asthma in the mother and maternal grandmother, rhinitis on the paternal side, and multiple allergies in the patient's sister. The maternal grandmother also has a history of diabetes mellitus and dyslipidemia. The father reportedly exhibits autistic traits. Vaccinations are up to date according to the patient's age and the regional immunization schedule.

Genetic evaluation via whole-exome sequencing identified a heterozygous pathogenic microdeletion at chromosome 15q11.2 (~252 kb, BP1-BP2 region), consistent with recurrent 15q11.2 microdeletion syndrome. Additionally, the patient is heterozygous for the pathogenic CFTR variant p.Phe508del, a class 1 mutation. A sweat chloride test via iontophoresis showed a sodium-chloride concentration of 54 mEq/L, slightly above the diagnostic threshold of 50 mEq/L, indicating a borderline result. However, the geneticist confirmed that this finding is not clinically significant given the patient’s carrier status for the CFTR mutation. Laboratory results obtained two months after the last brain tumor resection showed elevated eosinophil count and percentage, as well as increased immunoglobulin E (IgE) levels, consistent with the patient's history of atopy. Low valproic acid levels were attributed to instructions from the neurologist to withhold the medication the day before and on the day of testing to avoid interference. Detailed laboratory values are presented in Tables 1-2.

**Table 1:** Complete blood count WBC: white blood cell count; RBC: red blood cell count; MCV: mean corpuscular volume; MCH: mean corpuscular hemoglobin; MCHC: mean corpuscular hemoglobin concentration; RDW-CV: red blood cell distribution width-coefficient of variation; MPV: mean platelet volume * Clinically relevant abnormalities; # absolute count

Test name	Result	Reference range
WBC	8.41	5.00-14.50 10³ cel./µL
RBC	4.85	4.63-6.08 10⁶/µL
Hemoglobin	13.7	10.7-14.7 g/dL
Hematocrit	39.5	31.0-43.0%
MCV	81.4	79.0-92.2 fL
MCH	28.2	25.7-32.2 pg
MCHC	34.7	32.3-36.5 g/dL
Platelets	361	150-400 10³ cel./L
RDW-CV	12.9	11.6- 14.4%
MPV	10.5	9.4-12.4 fL
Lymphocytes #	3.07	1.32-3.57 10³ cel./µL
Lymphocytes (%)	36.5	21.8-53.1%
Neutrophils #	3.48	1.78-5.38 10³ cel./µL
Neutrophils (%)	41.4	34.0-67.9%
Monocytes #	0.61	0.30-0.82 10³ cel./µL
Monocytes (%)	7.3	5.3-12.2%
Basophils #	0.06	0.01-0.08 10³ cel./µL
Basophils (%)	0.7	0.2-1.2%
Eosinophils #	1.19*	0.04-0.8 10³ cel./µL
Eosinophils (%)	14.1*	0.8-7%

**Table 2:** Other relevant laboratory results ALT: alanine aminotransferase; AST: aspartate aminotransferase; Free T4: free thyroxine; Total T3: total triiodothyronine; Total T4: total thyroxine; TSH: thyroid stimulating hormone; IgE: immunoglobulin E * Clinically relevant abnormalities

Test name	Result	Reference range
ALT	13.0	0.0-50.0 U/L
AST	31.0	15.0-60.0 U/L
Serum iron	100.0	33.0-193.0 µg/dL
Valproic acid	47.10*	50.00-100.00 µg/mL
Free T4	1.21	0.93-1.70 ng/dL
Total T3	1.82	0.80-2.00 ng/mL
Total T4	7.88	5.99-13.8 µg/dL
TSH	2.52	0.60-4.84 µUI/mL
IgE	1430.0*	0-90 UI/mL

Currently, the patient is managed with antiepileptic and psychiatric medications, VNS, and multidisciplinary therapies, including speech and behavioral therapies. The patient’s antiepileptic medications include lacosamide 50 mg, administered as 1/2 tablet in the morning and one tablet in the afternoon; levetiracetam oral solution (100 mg/mL), 3 mL (300 mg) every 12 hours; aripiprazole oral solution (1 mg/mL), 1 mL (1 mg) every morning and 2 mL (2 mg) each afternoon; and valproic acid oral solution (100 mg/mL), 5 mL (500 mg) every 12 hours.

The patient's psychiatric medication includes clonidine for agitation and aggression, administered in divided doses totaling 0.2 mg daily: 0.05 mg (½ tablet) at 8:00 a.m., 0.025 mg (¼ tablet) at 12:00 p.m., 0.025 mg (¼ tablet) at 4:00 p.m., and 0.1 mg (1 tablet) at 9:00 p.m. Hydroxyzine is prescribed for insomnia, given as 10 mg (1 mL of oral solution, 10 mg/mL) at bedtime. Risperidone is prescribed to manage aggression and self-injurious behaviors, with a total daily dose of 0.75 mg, administered as 0.25 mg (0.25 mL, approximately five drops) in the morning and 0.5 mg (0.5 mL, approximately 10 drops) at night.

## Discussion

The 15q11.2 microdeletion syndrome is a rare genetic disorder caused by a missing portion of the long arm of chromosome 15, specifically in the 11.2 region, which contains five breakpoints (BP1-BP5). The inheritance pattern is autosomal dominant with incomplete penetrance and phenotypic variability [1,6]. The deletion in the BP1-BP2 region is found in only 0.5%-1% of the population [2]. The genetic material in this region encodes the following genes: non-imprinted in Prader-Willi/Angelman syndrome region protein 1 (NIPA1), non-imprinted in Prader-Willi/Angelman syndrome region protein 2 (NIPA2), cytoplasmic FMR1 interacting protein 1 (CYFIP1), and tubulin gamma complex component 5 (TUBGCP5) [7-9]. NIPA1 is involved in magnesium transport and is highly expressed in the brain. NIPA2 has been associated with childhood absence seizures. CYFIP1 stabilizes axon processes and dendritic complexity, while TUBGCP5 has been linked to ADHD and obsessive-compulsive disorder (OCD) [1]. These genes are related to nervous system function, which explains why this syndrome may present with various central nervous system (CNS) manifestations, such as developmental delay, neurobehavioral issues (including ADHD, ASD, dyslexia, psychosis, schizophrenia, and OCD), abnormal brain imaging, seizures, spastic paraplegia and congenital abnormalities, for example, dysmorphic features [3,7,10].

Patients with BP1-BP2 microdeletion often have mild or no clinical symptoms (seen in approximately 90% of cases), and the variability in manifestations can complicate diagnosis and genetic counseling. Because the affected region is so small, standard karyotyping cannot detect it; instead, CMA is used for diagnosis and can aid in genetic counseling for prospective parents. Prenatal diagnosis using ultrasound has also been attempted; however, only 44% of fetuses with 15q11.2 microdeletions showed abnormalities (e.g., cardiac and vascular defects, elevated nuchal translucency, brain structural abnormalities, intrauterine growth restriction, skeletal anomalies, etc.) [1,11,12]. Still, our patient didn't present with prenatal ultrasound abnormalities and exhibited a rare neonatal onset of symptoms, comparable to case reports describing unprovoked generalized tonic-clonic seizures emerging as early as six months of age [13].

Treatment for individuals with BBS is personalized and typically involves comprehensive evaluation by geneticists, psychiatrists, psychologists, neuropsychologists, and developmental experts. Management may include educational interventions, various therapies, and surgical or dental treatments. Magnesium supplementation has been suggested because two of the four genes involved in BBS are implicated in magnesium transport, leading to spastic paraplegia and absence epilepsy, although evidence supporting its use is limited [7]. Regular assessments are crucial, as the pathology may worsen with age [1,7]. Early intervention in children with developmental disorders is also essential, as it leads to improved outcomes [14].

## Conclusions

This case of a six-year-old male with a 15q11.2 microdeletion highlights the extensive clinical variability and complexity associated with this genetic syndrome. We report novel aspects such as the early-onset schizencephaly, the CFTR carrier status with borderline sweat chloride test, and the presence of multiple allergic and behavioral comorbidities, which underscores the importance of early genetic evaluation. The identification of a pathogenic BP1-BP2 deletion via chromosomal microarray allowed for a more comprehensive understanding of the patient's neurodevelopmental and medical challenges. This understanding was crucial in planning personalized care and providing family counseling. Given the often subtle and non-specific clinical features, it's essential for clinicians to have greater awareness to ensure timely diagnosis and management. This case supports the integration of genetic testing in the evaluation of children with neurodevelopmental disorders and complex multisystemic manifestations. While the 15q11.2 microdeletion is strongly associated with neurodevelopmental and epileptic features, the extensive multisystem involvement observed in this patient, such as early-onset schizencephaly, type 2 diabetes, asthma, and CFTR-related findings, suggests the contribution of additional genetic, environmental, or epigenetic factors. A more integrative interpretation may be required to fully explain the phenotype, particularly in the context of coexisting variants such as the CFTR carrier status.
